# Extracellular ATP Induced S-Phase Cell Cycle Arrest via P2Y Receptor-Activated ERK Signaling in Poorly Differentiated Oral Squamous Cell Carcinoma SAS Cells

**DOI:** 10.3390/life11111170

**Published:** 2021-11-02

**Authors:** Chia Chih Lau, Amnani Aminuddin, Kok Meng Chan, Ian C. Paterson, Lok Mun Law, Pei Yuen Ng

**Affiliations:** 1 Centre for Drug and Herbal Development, Faculty of Pharmacy, Universiti Kebangsaan Malaysia, Kuala Lumpur 50300, Malaysia; cclau92@gmail.com (C.C.L.); amnani.aminuddin@gmail.com (A.A.); lawlokmun@gmail.com (L.M.L.); 2 Center for Toxicology and Health Risk Studies, Faculty of Health Sciences, Universiti Kebangsaan Malaysia, Kuala Lumpur 50300, Malaysia; chan@ukm.edu.my; 3 Department of Oral & Craniofacial Sciences, Faculty of Dentistry, Universiti Malaya, Kuala Lumpur 50603, Malaysia; ipaterson@um.edu.my

**Keywords:** extracellular ATP, oral squamous cell carcinoma, P2Y receptors, ERK signaling, S-phase arrest

## Abstract

Extracellular ATP in the tumor microenvironment exhibits either pro- or antitumor effect via interaction with P2Y receptors, but the intracellular signaling and functional roles of P2Y receptors in oral squamous cell carcinoma (OSCC) are unclear. We aimed to study the effect of ATP on OSCC cell lines and the potential mechanisms involved. Through GEPIA dataset analysis, high expression levels of mRNA encoding P2Y receptors, the ATP-induced G protein-coupled receptors, were associated with better overall patient survival in head and neck squamous cell carcinoma. qPCR analysis showed that the poorly differentiated OSCC SAS cell line, had higher *P2RY1* expression level compared to the well-differentiated H103 and H376 cell lines. Western blotting and flow cytometry analyses revealed that ATP phosphorylated ERK and elevated intracellular calcium signaling in all tested cell lines. A significant S-phase cell cycle arrest was observed in SAS, and preincubation with the MEK inhibitor PD0325901 reversed the ATP-induced S-phase arrest. We further demonstrated that ATP induced a slight reduction in cell count and colony formation yet significant apoptosis in SAS. Overall, we postulate that the ATP-induced S-phase arrest effect in SAS cells may be regulated through P2Y receptor-mediated ERK signaling, thus suggesting a potential antitumor effect of ATP via interaction with its distinct profile of P2Y receptors.

## 1. Introduction

Oral squamous cell carcinoma (OSCC) is one of the common malignant types of head and neck cancers and accounts for 3% of malignant tumors worldwide [1]. Despite advancement in therapeutic interventions including adjuvant therapies with radiotherapy, systemic chemotherapy, and/or topical chemotherapy, an overall improvement on the 5-year survival rate in OSCC patients is still limited due to the aggressive local invasion and highly metastatic profile of OSCC [2,3,4,5]. Multiple cohort studies revealed that up to 40% of the patients experienced disease recurrence with increased tumor invasiveness within two years after completion of treatment [6,7,8]. Chemotherapy is the common choice of treatment of advanced OSCC for an overall improvement in the survival rate among patients. However, its effectiveness is often hampered by the development of drug resistance [9,10]. The development of an alternative treatment approach is therefore crucial for improving the overall survival rate and the quality of life among patients.

Adenosine 5′-triphosphate (ATP) is a key extracellular signaling molecule that couples to specific purinergic receptors to mediate various biological responses including signal transmission, proliferation, differentiation, and cell death [11]. To execute those responses, ATP can act on two different types of G protein-coupled receptors, namely iono atropic P2X and metabotropic P2Y receptors [12]. P2Y receptors are widely expressed in various tissues, while P2X receptor expression is restricted to the nervous system, platelets, and smooth muscle cells [13]. P2Y receptors are further subdivided into eight different subtypes, and most of them are coupled with G_q/11_ protein [14]. Upon activation, phosphatidylinositol bisphosphate (PIP_2_) is hydrolyzed to inositol 1,4,5-triphosphate (IP_3_) to trigger the release of calcium ion (Ca^2+^) from the endoplasmic reticulum into cytosol. The increase in cytosolic Ca^2+^ regulates almost all cellular events ranging from proliferation to apoptosis [15].

Under normal physiological conditions, ATP is mainly located intracellularly (~5–10 mM) and is scarcely found in the extracellular compartment. Intriguingly, extracellular ATP has been detected in the micromolar range in the tumor microenvironment [16,17,18]. It has been reported that tumor cell death due to hypoxia, inflammation, or anticancer therapies promotes an increase in the extracellular ATP [19]. Accumulating evidence has shown that the release of ATP and subsequent activation of P2Y receptors contribute to a vast array of cellular responses in tumor cells ranging from proliferation to apoptosis in different types of cancers [14]. Among the P2Y receptor subtypes, P2Y_1_, P2Y_2,_ and P2Y_11_ receptors are frequently studied in cancers [20]. For example, the potential role of P2Y_1_ and P2Y_2_ receptors in the regulation of cancer progression and pathogenesis of OSCC has been reported based on gene expression analysis [21,22]. On the other hand, it has been reported that P2Y_1_ activation induces apoptosis in prostate and gastric cancer [23,24]. In another study, the activation of P2Y_11_ inhibited the migration of tumor-derived endothelial cells [25].

Among the associated signaling pathways of G protein-coupled receptors, the extracellular signal-regulated kinase (ERK) pathway is one of the major pathways regulated by P2Y receptors for the regulation of cell proliferation and survival [26,27]. P2Y receptor-mediated ERK activation has been reported to promote cancer progression in esophageal, breast, and prostate cancer [28,29,30]. In contrast, the activation of ERK by P2Y receptors promoted an antiproliferative effect in colorectal and intestinal cancer [31,32]. In head and neck squamous cell carcinoma (HNSCC), it has been reported that P2Y_2_ receptor-activated ERK1/2 signaling promotes proliferation of CAL27 cells, and further blockade of the P2Y signaling via a specific antagonist and genetic knockout reduced UTP-induced CAL27 proliferation *in vitro* [33]. Overexpression of ERK has been well observed in OSCC, though the correlation of P2Y receptor activation with ERK in OSCC has yet to be understood [34,35].

Hence, this present study aimed to study the expression and functional role of the P2Y receptors in OSCC. In this context, this study particularly aimed to investigate the effect of P2Y receptor-mediated ERK activation by ATP in OSCC. Understanding the potential mechanisms by which P2Y receptors promote the development and progression of OSCC may facilitate the development of more effective targeted therapy for improving patient survival.

## 2. Materials and Methods

### 2.1. Cell Culture

Two human OSCC cell lines, namely H103 and H376, originating from the tongue and the floor of the mouth, respectively, were derived and cultured as described previously [36]. An additional OSCC cell line, SAS, originating from the tongue, was purchased from the Japanese Collection of Research Bioresources (JCRB) Cell Bank (Osaka, Japan). All three cell lines originated from different cancer stages, and their detailed characteristics are provided in Table S1. Cells were grown and maintained in a humidified incubator at 37 °C with 5% CO_2_ atmosphere, in the DMEM-F12 medium (Nacalai Tesque, Kyoto, Japan) supplemented with 100 units/mL penicillin–streptomycin (Nacalai Tesque, Kyoto, Japan) and 10% fetal bovine serum (Hyclone, Logan, UT, USA). For both H103 and H376, an additional 0.5 µg/mL sodium hydrocortisone succinate (Sigma Aldrich, St Louis, MI, USA) was added to the DMEM-F12 growth medium.

### 2.2. GEPIA Dataset Analysis

An online collection of RNA sequencing expression data of 9736 tumors and 8587 normal samples from The Cancer Genome Atlas (TCGA) and Genotype-Tissue Expression (GTEx) projects, namely Gene Expression Profiling Interactive Analysis (GEPIA), was used to analyze the differential expression levels of the P2Y receptors across cancers, including HNSCC, and their patient survival analysis (Accessed on 22 October 2021, http://gepia.cancer-pku.cn/) [37].

### 2.3. Real-Time Polymerase Chain Reaction (qPCR)

qPCR was performed to measure the gene expression levels of receptor subtypes, namely *P2RY1*, *P2RY2*, and *P2RY11*. Total RNA was extracted using an InnuPrep RNA Mini Kit (Analytik Jena, Jena, Denmark) and converted to cDNA with a Tetro cDNA Synthesis Kit (Bioline, Cincinnati, OH, USA). qPCR was performed on a CFX Connect Real-Time PCR Detection System (Bio-Rad Laboratories Inc., Hercules, CA, USA) using a SensiFast SYBR No-Rox Kit (Bioline, Cincinnati, OH, USA) with an initial denaturation step of 95 °C for 2 min, followed by 45 cycles of 95 °C for 5 s, 65 °C for 10 s, and 72 °C for 20 s. An additional postamplification melting curve step was performed subsequently. The gene expression levels were normalized to those of reference genes, tubulin alpha-6 chain (*TUBA6*) and ribosomal protein S13 (*RPS13*). The primers used are listed in Table S2.

### 2.4. Measurement of Intracellular Ca^2+^

The level of Ca^2+^ release into the cytosol upon ATP treatment was measured using a flow cytometric analysis following staining with a cell-permeable calcium-binding fluorescent dye, Fluo 2 LeakRes AM (Abcam, Cambridge, UK). Briefly, 1 × 10^5^ cells were collected and resuspended in 1 mL of cold phosphate-buffered saline (PBS) containing 4 µM Fluo 2 LeakRes AM. The basal fluorescence intensity was recorded for 1 min at 10 s intervals on a BD FACSCanto II Cell Analyzer (BD Biosciences, San Jose, CA, USA) with an excitation wavelength at 490 nm. Subsequently, a final concentration of 100 µM ATP (Sigma Aldrich, St Louis, MI, USA) was added into each sample, and the fluorescence intensity was recorded immediately at 10 s intervals for 4 min. Results obtained were analyzed using BD FACSDiva Software version 6.1.3 (BD Biosciences, San Jose, CA, USA), and a graph of fold increase in intracellular Ca^2+^ level against time was plotted.

### 2.5. Western Blotting

The phosphorylation levels of ERK in ATP-treated cell lines were measured via Western blotting. Briefly, cells were seeded on a 12-well plate at a density of 1 × 10^4^ cells/well and incubated at 37 °C with 5% CO_2_. Upon reaching 70% confluency, cells were treated with 100 µM ATP at different time points (5–120 min). After the treatment, cells were lysed with 1× Laemmli buffer. Equal amounts of protein were separated electrophoretically by 10% sodium dodecyl sulfate–polyacrylamide gels at 200 V for 100 min and were transferred to nitrocellulose membranes (GE Healthcare Life Sciences, Chicago, IL, USA) at 100 V for 75 min. The membranes were then blocked with 5% (*w*/*v*) nonfat skim milk for 1.5 h. The membranes were separately probed with the primary antibodies against the rabbit monoclonal phosphorylated p44/42 MAPK and p44/42 MAPK antibodies (1:1000; Cell Signaling Technology, Danvers, MA, USA) at room temperature overnight. After the primary antibody incubation, the membranes were washed with a washing buffer containing 150 mM NaCl, 20 mM Tris, and 0.03% (*v*/*v*) Tween-20 (NaTT buffer). Further, the membranes were incubated with the secondary anti-rabbit monoclonal antibody (1:3000; Cell Signaling Technology, Danvers, MA, USA) for 2 h at room temperature. The protein bands were visualized using enhanced chemiluminescence (ECL) substrates on ChemiDoc XRS+ (Bio-Rad Laboratories Inc., Hercules, CA, USA) and were quantified by a densitometry analysis using Image Lab 5.2.1 (Bio-Rad Laboratories Inc., Hercules, CA, USA). The raw outputs of the protein bands and densitometry readings are provided in Figures S1–S3 and Tables S3–S5, respectively.

### 2.6. Cell Cycle Assay

The cell cycle assay was carried out by using flow cytometry. In brief, cells were seeded at 1 × 10^6^ cells/well on a 6-well plate and incubated at 37 °C with 5% CO_2_. Cells were treated with test compounds (ATP, ATPγS, adenosine, suramin, PD0325901, or docetaxel) at varying concentrations or time points (24–72 h or 24 h alone). Cell pellets were then harvested by trypsinization and fixed with 70% (*v*/*v*) cold ethanol at −20 °C overnight. Prior to analysis, cell pellets were washed twice with cold PBS and incubated in a staining solution containing 50 µg/mL propidium iodide (PI) and 0.1% (*v*/*v*) Triton X for 30 min at room temperature. The fluorescence intensity was measured using BD FACSCanto II Cell Analyzer (BD Biosciences, San Jose, CA, USA), and the proportion of cells in G1, S, and G2/M phases was analyzed using Mod Fit LT 2.0 software (Verity Software House Inc., Topsham, ME, USA).

### 2.7. Trypan Blue Exclusion Assay

The viability of cells after ATP treatment was measured and compared against the untreated cell control using a trypan blue exclusion assay. In brief, cells were seeded on a 6-well plate with a total of 0.5 × 10^3^ cells/well and incubated at 37 °C with 5% CO_2_ for 24 h. Cells were then washed with PBS, and the medium was replaced with a new medium. Then, cells were treated with ATP at a final concentration of 100 µM at different time points (24–72 h). Cells were then trypsinized and resuspended with 1 mL of PBS. A total of 10 µL of each cell suspension was mixed with 10 µL of 0.4% trypan blue (Sigma Aldrich, St Louis, MO, USA) and loaded onto a cell counting slide (Bio-Rad Laboratories Inc., Hercules, CA, USA). The cell number was quantified using a TC10 automated cell counter (Bio-Rad Laboratories Inc., Hercules, CA, USA).

### 2.8. Clonogenic Assay

Similar cell seeding and stimulation conditions were employed as described in the trypan blue exclusion assay section. After the treatment with ATP, cells on the cultured plate were washed with PBS and fixed with 2 mL of methanol for 30 min. Methanol was removed and cells were stained with 5 mL of 0.01% (*w*/*v*) crystal violet for 30 min. Each well plate was rinsed with distilled water to remove excess stain. The cultured plate was left to dry overnight at room temperature. The colony images were captured using ChemiDoc XRS+ (Bio-Rad Laboratories Inc., Hercules, CA, USA) with white light transilluminator. Java-based image processing program ImageJ (National Institutes of Health, Bethesda, MD, USA) was used to quantify the number, area, and intensity of the colonies. A colony was defined by a cell cluster containing more than 50 cells.

### 2.9. Apoptosis Assay

To measure the percentage of viable, apoptotic, or necrotic cells, cells were stained using Annexin V–fluorescein isothiocyanate conjugate (FITC) and PI Apoptosis Kit (BD Biosciences, San Jose, CA, USA) according to the manufacturer’s instructions. Similar cell seeding and ATP stimulation conditions were employed as described in the cell cycle assay section. After the treatment, cells were harvested by trypsinization, resuspended in 100 µL of 1× binding buffer, and stained with 5 µL of Annexin V–FITC and 5 µL of PI. Cells were incubated for 15 min in a dark room. A total of 400 µL of 1× binding buffer was added prior to a flow cytometric analysis using BD FACSCanto II Cell Analyzer (BD Biosciences, San Jose, CA, USA).

### 2.10. Statistical Analysis

All data are presented as mean and standard error of mean (SEM) of three independent experiments. Data were statistically analyzed using one-way analysis of variance (ANOVA) with Bonferroni’s multiple comparison post hoc test via GraphPad Prism version 5.0 (GraphPad Software, Inc., San Diego, CA, USA). Differences between groups were considered statistically significant when *p* < 0.05.

## 3. Results

### 3.1. Expression and Patient Survival Analysis of P2Y Receptors in HNSCC

**Figure 1:**
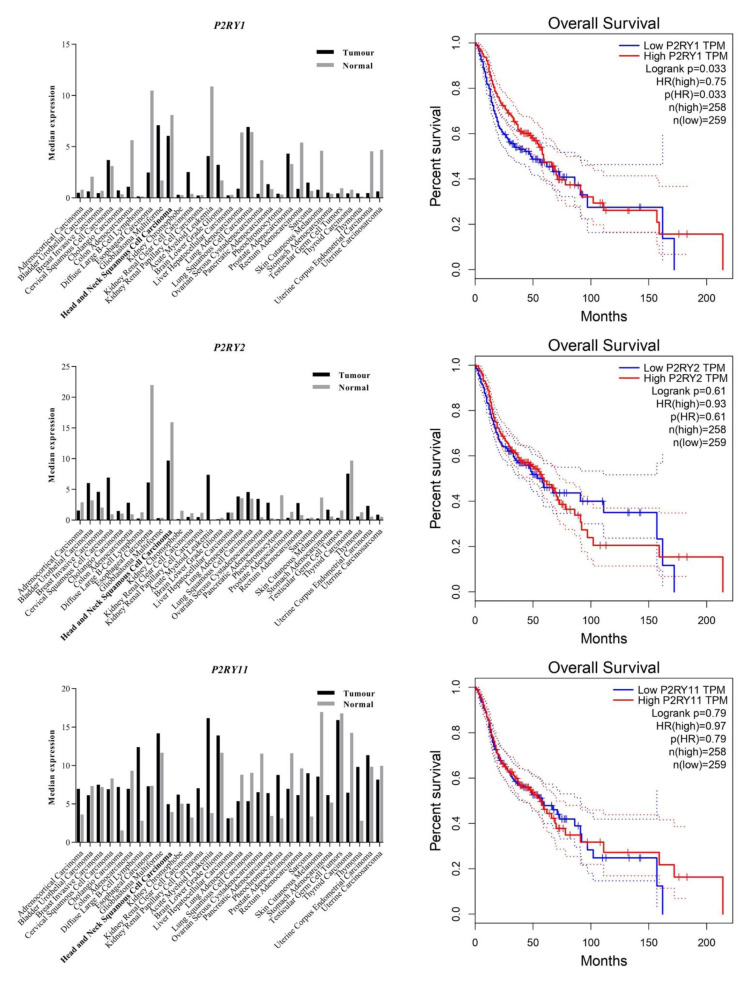
Gene expression and patient survival analysis of P2Y receptors across cancers and normal tissues. The data were extracted from the GEPIA datasets which consist of RNA sequencing expression data from the TCGA and GTEx projects [37].

As illustrated in Figure 1, the expression data analysis from GEPIA demonstrated a significant downregulation of *P2RY1* and *P2RY2* in HNSCC compared to normal tissues. Low expression levels of *P2RY1* and *P2RY2* were associated with reduced overall patient survival. On the other hand, HNSCC samples expressed slightly higher *P2RY11* compared to the normal tissues, and its high expression was associated with longer survival. The varied findings on the expression levels of the receptor subtypes and the disease prognosis possibly suggest the complex roles of the cancer-specific landscape of P2Y receptors in HNSCC.

### 3.2. Expression of mRNA Encoding P2Y Receptors in OSCC Cell Lines

**Figure 2:**
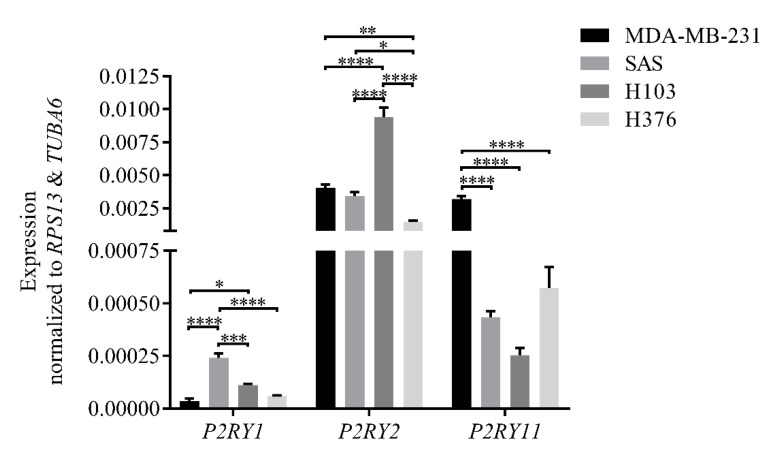
The expression levels of mRNA encoding P2Y_1_, P2Y_2_, and P2Y_11_ receptors in OSCC cell lines normalized to those of the reference genes, *TUBA6* and *RPS13*, as analyzed by qPCR. A highly P2Y_2_ receptor-expressing MDA-MB-231 cell line was used as a technical positive control. Data are presented as mean ± SEM of three independent experiments, * *p* < 0.05, ** *p* < 0.01, *** *p* < 0.001, **** *p* < 0.0001.

In this study, the expression of mRNA encoding P2Y_1_, P2Y_2_, and P2Y_11_ receptors in H103, SAS, and H376 cell lines was profiled using qPCR. In the analysis, a highly P2Y_2_ receptor-expressing human breast adenocarcinoma cell line, namely MDA-MB-231, was used as the technical positive control [38]. As illustrated in Figure 2, SAS had the highest expression of *P2RY1*, followed by H103 and H376. Meanwhile, H103 expressed *P2RY2* in abundance, followed by SAS and H376. All three OSCC cell lines, however, showed low expression of *P2RY11* when compared to MDA-MB-231. Comparing between three different OSCC cell lines, the findings significantly demonstrated that the advanced stage cancer cell lines had lower expression of the gene encoding the P2Y_2_ receptor. Meanwhile, the poorly differentiated SAS cells had higher *P2RY1* expression compared to the well-differentiated H103 and H376 cells, thus reflecting the difference in the expression levels of receptor subtypes based on the differentiation state of the cells.

### 3.3. ATP Induced Intracellular Ca^2+^ Release and ERK Phosphorylation in OSCC Cell Lines

**Figure 3:**
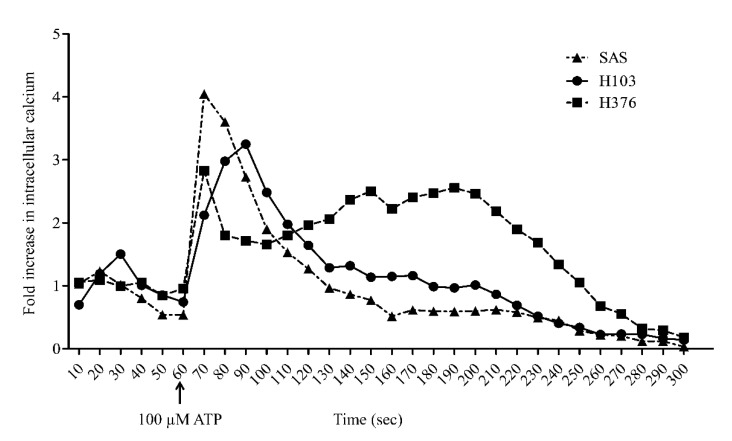
The activation of G_q/11_-coupled P2Y receptors through ATP treatment as measured by the increased level of cytosolic free Ca^2+^ using flow cytometry. Basal level of cytosolic Ca^2+^ was captured in the first minute prior to treatment with 100 µM ATP. The transient intracellular Ca^2+^ release in OSCC cell lines was measured within 4 min after ATP treatment.

In this study, the activation of G_q/11_-coupled P2Y receptors through ATP treatment was indicated by the elevated level of cytosolic free Ca^2+^. The addition of 100 µM ATP at 60 s rapidly induced a peak increase in intracellular Ca^2+^ concentration up to 4-fold within 10 s in SAS cells (Figure 3). By contrast, H103 showed a gradual increase in Ca^2+^ concentration and only peaked at 90 s. Both SAS and H103 showed a 50% reduction in Ca^2+^ concentrations within 30 s before gradually returning to baseline at approximately 250 s. On the other hand, H376 showed a relatively lower peak increase in Ca^2+^ concentration when induced by ATP compared to SAS and H103. Interestingly, the Ca^2+^ concentration in H376 remained high for a longer duration before returning to baseline at 280 s.

**Figure 4:**
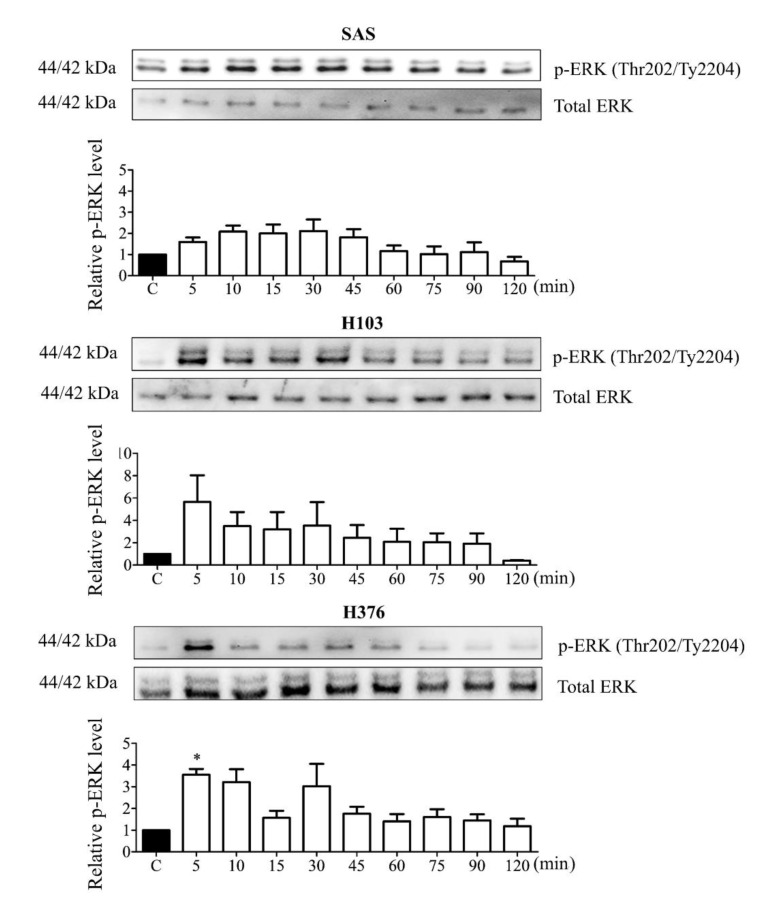
ATP induced transient ERK phosphorylation in OSCC cell lines. Cells were treated with 100 µM ATP for several time points and the ERK phosphorylation levels were measured by Western blotting. The p-ERK levels after ATP treatment were normalized against total ERK levels and compared with the untreated control group (labeled as C in the graph). Data are presented as mean ± SEM of three independent experiments, * *p* < 0.05.

The activation of G_q/11_-coupled P2Y receptors leads to ERK MAPK phosphorylation (p-ERK). From the Western blotting analysis, short-term phosphorylation of ERK was demonstrated in all three cell lines upon treatment with 100 µM ATP with a maximal phosphorylation level at 5 min for H103 and H376 and 15 min for SAS (Figure 4). After the maximal phosphorylation, the phosphorylation level in H103 decreased by half after 10 min before gradually being reduced. The ERK phosphorylation in H376 was the most transient as it dropped drastically after 15 min of ATP treatment. On the other hand, SAS demonstrated a later and lower peak level of ERK phosphorylation compared to H103 and H376, but the activity was prolonged before gradually being reduced. The differences in the magnitude and duration of intracellular Ca^2+^ release and ERK phosphorylation observed in the three cell lines could be attributed to the differences in the expression levels of P2Y receptor subtypes.

### 3.4. ATP Induced S-Phase Arrest via ERK Signaling

**Figure 5:**
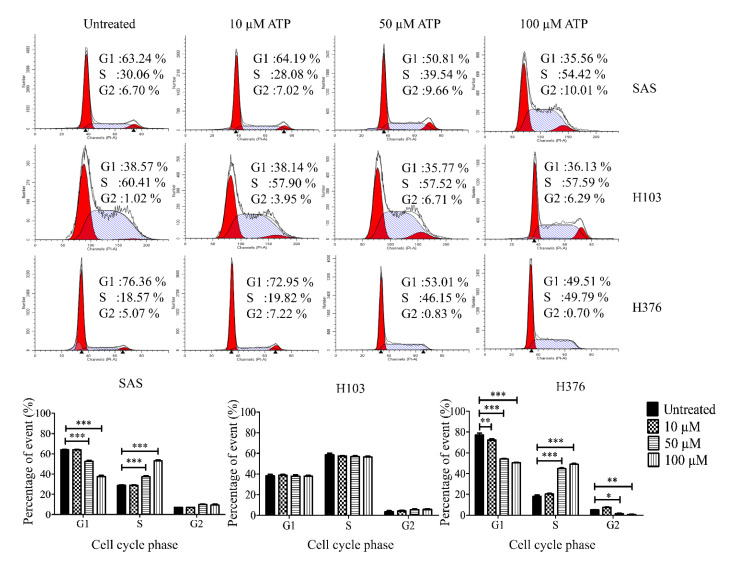
Effects of different concentrations of ATP treatment on the cell cycle phases in OSCC cell lines, as analyzed by flow cytometry. The representative histograms of the cell cycle phases and analysis of the percentage of cells in cell cycle phases after treatment with varying doses of ATP for 24 h compared to the untreated control group. Data are presented as mean ± SEM of three independent experiments, * *p* < 0.05, ** *p* < 0.01, *** *p* < 0.001.

**Figure 6:**
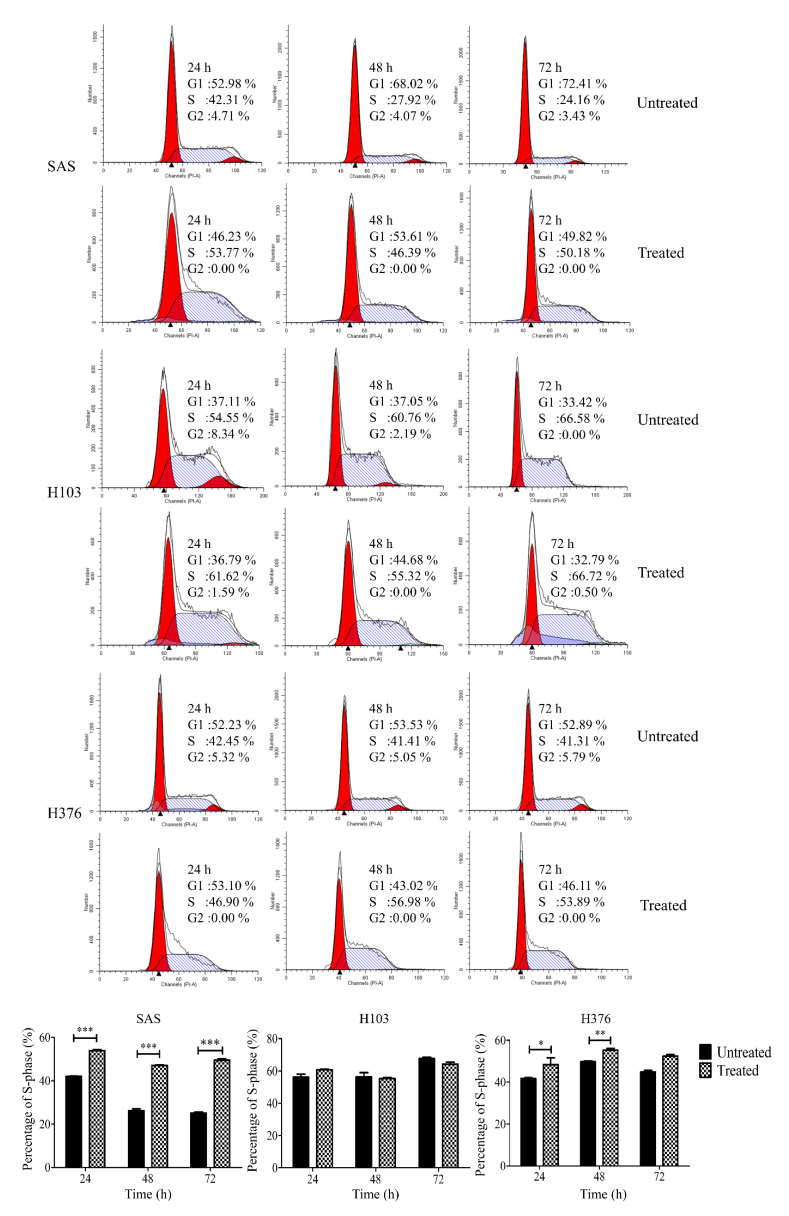
Effects of a high concentration of ATP treatment on the cell cycle phases in OSCC cell lines after different time points, as analyzed by flow cytometry. The representative histograms of the cell cycle phases and the percentage of S-phase cells for both treated and untreated control groups at 24–72 h. Data are presented as mean ± SEM of three independent experiments, * *p* < 0.05, ** *p* < 0.01, *** *p* < 0.001.

The conflicting pro- or antitumor role of P2Y receptors in cancers prompted us to evaluate the functional role of the receptors in OSCC upon ATP treatment. Firstly, we evaluated the effect of ATP in modulating the cell cycle. We found that ATP induced S-phase arrest in SAS and H376 in a dose-dependent manner after 24 h of treatment (Figure 5). Only a high concentration of ATP caused a significant increase in S-phase cells in SAS and H376, suggesting the potential biphasic effects of ATP in a dose-dependent manner. However, no changes in the cell cycle were observed in H103 cells at all concentrations tested. As shown in Figure 6, a high concentration of ATP induced a significant S-phase arrest in the advanced stage cancer cell lines SAS (at all tested time points) and H376 (at 24 and 48 h). Again, no significant changes in the cell cycle phases were observed in H103 cells upon ATP treatment, which is possibly due to the differences in the expression of different P2Y receptor subtypes between the cell lines.

**Figure 7:**
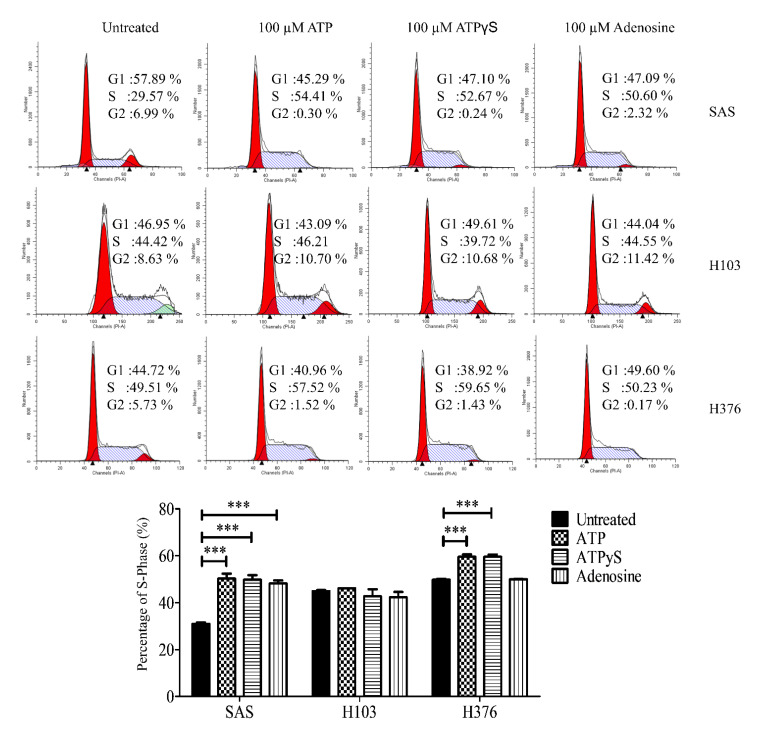
Effects of ATPγS, the nondegradable version of ATP, and adenosine treatments in inducing S-phase arrest in OSCC cell lines. The representative histograms of the cell cycle phases and analysis for the percentage of cells in the S phase in OSCC cell lines upon treatment with ATP, ATPγS, or adenosine for 24 h. Data are presented as mean ± SEM of three independent experiments, *** *p* < 0.001.

To rule out the possibility of S-phase arrest due to the degradation of ATP into ADP or adenosine, cells were also treated with a nondegradable version of ATP, namely ATPγS, and adenosine for 24 h. Similar findings were observed in all three cell lines after 24 h treatment with ATPγS when compared to the treatment with ATP (Figure 7). Meanwhile, adenosine at 100 µM only induced a significant S-phase arrest in SAS, while showing no effects in both H103 and H376.

**Figure 8:**
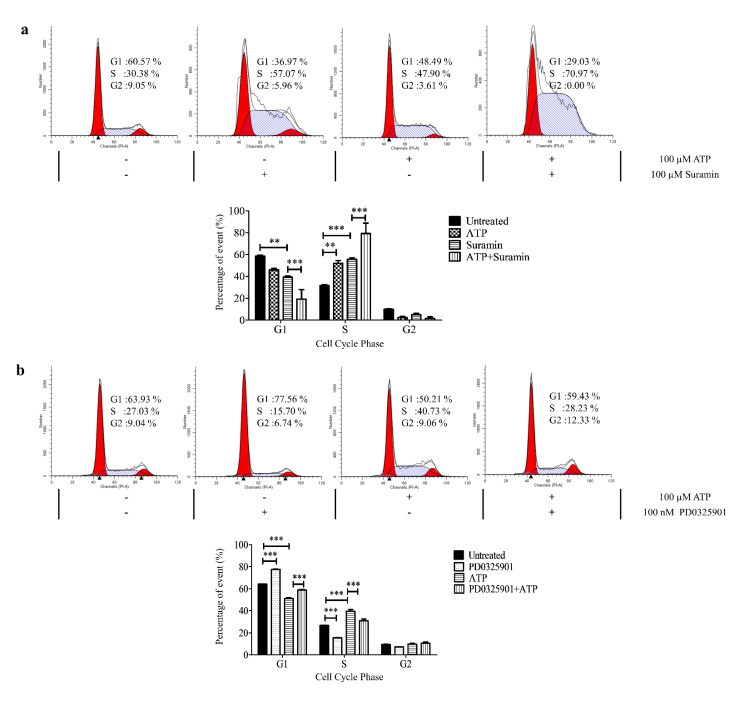
ATP induced S-phase arrest via P2Y receptor-mediated ERK signaling in SAS. (**a**) The representative histograms of the cell cycle phases and analysis of the percentage of cells in cell cycle phases after treatment with ATP alone, suramin alone, or ATP and suramin in combination for 24 h. (**b**) The representative histograms of the cell cycle phases and analysis of the percentage of cells in cell cycle phases after treatment with ATP alone, MEK inhibitor (PD0325901) alone, or ATP and MEK inhibitor in combination for 24 h. Data are presented as mean ± SEM of three independent experiments, ** *p* < 0.01, *** *p* < 0.001.

The direct effect of ATP on the P2Y receptors in inducing S-phase arrest in SAS particularly was evaluated by treating the cells with a nonspecific purinergic receptor antagonist, suramin [39,40,41]. As shown in Figure 8a, suramin caused an increase in S-phase arrest at a similar magnitude observed in cells treated with ATP. Meanwhile, a preincubation of suramin followed by ATP treatment synergistically caused a tremendous increase in S-phase arrest in SAS. In this study, a direct correlation of the action of ATP on P2Y receptors to modulate the cell cycle arrest cannot be elucidated using suramin intervention.

This study further investigated whether the inhibition of the upstream of ERK activator using a MAPK/ERK kinase (MEK) inhibitor, PD0325901, could reverse the ATP effect in inducing S-phase arrest in SAS. As shown in Figure 8b, incubation with 100 nM PD0325901 alone significantly reduced the percentage of cells in the S phase compared to untreated cells. A combination of ATP and PD0325901 treatment demonstrated a significant reduction in the percentage of cells in the S phase when compared to the cells with ATP treatment alone, thus suggesting a significant reversal of the ATP effect in inducing S-phase arrest via ERK activation through MEK inhibitor intervention.

### 3.5. ATP Suppressed Cell Proliferation and Significantly Induced Apoptosis in OSCC Cell Lines

**Figure 9:**
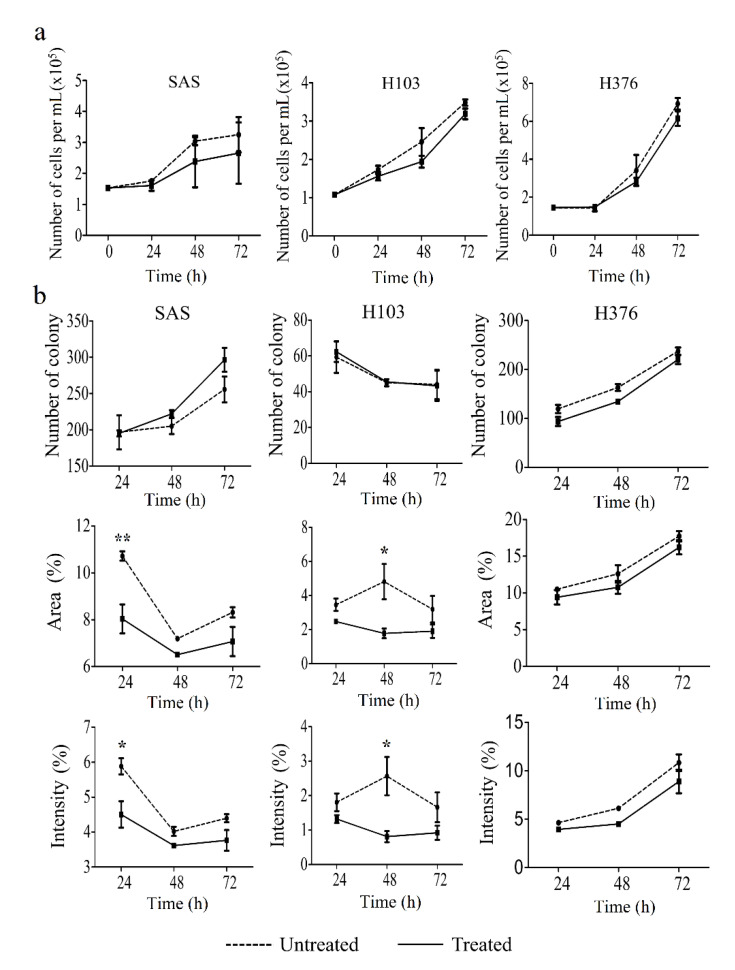
Effect of ATP treatment on the cell viability and proliferation of OSCC cell lines, as analyzed by trypan blue exclusion and clonogenic assays, respectively. (**a**) Cell viability was evaluated at several time points for 72 h after treatment with ATP. (**b**) The number and the percentage of area and intensity of the colonies after 24, 48, and 72 h treatment with ATP in OSCC cell lines. Data are presented as mean ± SEM of three independent experiments, * *p* < 0.05, ** *p* < 0.01.

To confirm the effect of ATP in inducing S-phase arrest, and therefore cellular apoptosis, trypan blue exclusion, clonogenic, and apoptosis assays were carried out. A high concentration of ATP treatment for 24, 48, and 72 h caused a slight reduction in cell viability of OSCC cell lines when compared to untreated cells, as evaluated using trypan blue exclusion assay, though statistically insignificant (Figure 9a). Correspondingly, the clonogenic analysis similarly demonstrated a reduction in the area and intensity of the colonies in ATP-treated cells compared to their respective untreated cells (Figure 9b). However, the findings for colony number were inconsistent in SAS and H103, presumably due to variation in size between colonies (Figure 9b).

**Figure 10:**
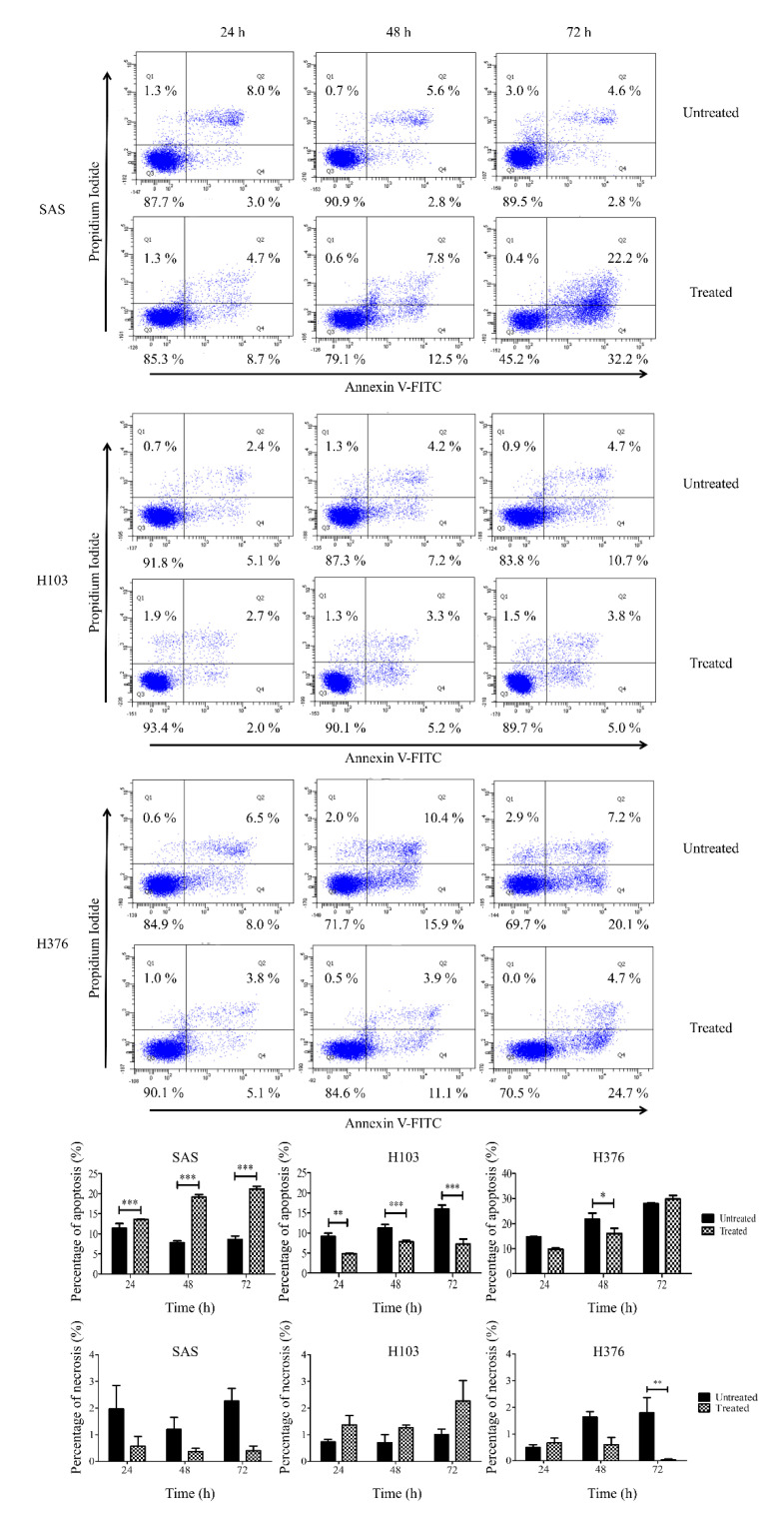
Effects of ATP treatment in inducing apoptosis in OSCC cell lines, as analyzed by flow cytometry. The representative diagrams of viable, apoptotic, or necrotic cells and analysis for the percentage of apoptotic and necrotic cells in OSCC cell lines for both treated and untreated control groups at 24–72 h. Data are presented as mean ± SEM of three independent experiments, * *p* < 0.05, ** *p* < 0.01, *** *p* < 0.001.

Further apoptosis assay demonstrated that the treatment of SAS with ATP caused a significant increase in the percentage of apoptotic cells (Figure 10). Corresponding to the cell cycle analysis, a significant S-phase arrest in SAS cells (Figure 6) significantly led to cellular apoptosis, and therefore the cell count and colony formation were reduced (Figure 9). Nevertheless, a significant reduction in apoptosis was observed in H376 at 48 h alone (Figure 10), despite a significant S-phase arrest being demonstrated after ATP treatment at both 24 and 48 h (Figure 6). Surprisingly, H103 showed a significant reduction in apoptosis at all tested time points (Figure 10), though no changes in the cell cycle phases were observed (Figure 6). These findings indicated that ATP induced S-phase arrest and subsequently induced apoptosis in SAS cells in particular.

### 3.6. ATP Did Not Influence the Mitotic Arrest Effect of Docetaxel

**Figure 11:**
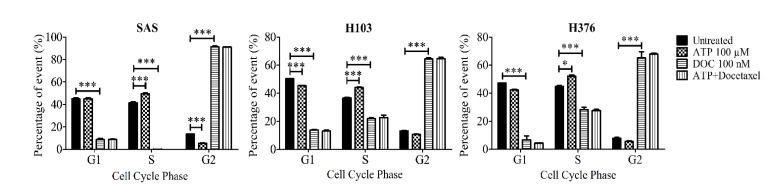
Effect of the combination treatment of ATP with docetaxel, an antimitotic chemotherapeutic agent, on the cell cycle phases after 24 h, as analyzed by flow cytometry. Data are presented as mean ± SEM of three independent experiments, * *p* < 0.05, *** *p* < 0.001.

Docetaxel, a semisynthetic taxane, is one of the chemotherapeutic agents used in the treatment of OSCC. It induces its cytotoxic effect by causing mitotic arrest at the G2/M phase. It has also been reported to induce a secondary cytotoxic effect via targeting S-phase cells [42]. The earlier findings of this study had demonstrated that the ATP treatment significantly promoted the S-phase arrest state of SAS and H376 particularly. Hence, this present study further investigated whether the combination treatment of ATP with docetaxel could promote a synergistic or antagonistic mitotic arrest effect of docetaxel in OSCC cell lines. Cells were first pretreated with 100 µM ATP for 30 min prior to treatment with 100 nM docetaxel for 24 h. As shown in Figure 11, docetaxel alone caused a significant reduction in the number of S-phase cells and promoted an increase in the G2/M-phase cells in all three cell lines. However, there was no difference in the proportion of G2/M-phase cells in the cells treated with both ATP and docetaxel when compared to the cells treated with docetaxel alone. This finding suggests that ATP did not induce any synergistic or inhibitory effect of docetaxel in OSCC cell lines.

## 4. Discussion

In this study, we utilized three OSCC cell lines with different tumor stages and clinicopathological characteristics to study the effect of extracellular ATP on OSCC cell lines with differential P2Y receptor profiles. The complexity of the receptors’ role across cancers makes it tricky to directly extrapolate the information on other cancers across understudied cancers, particularly OSCC. The contradicting roles of P2Y receptors in different cancers could be due to their up- or downregulated expression of receptor subtypes compared to the normal tissues, thus forming a cancer-specific landscape of P2Y receptors in the cancer cells. From the GEPIA dataset analysis, the downregulation of genes encoding P2Y_1_ and P2Y_2_ receptors is common in HNSCC—where OSCC is one of the common subsets of HNSCC—compared to the normal tissues. The high expression of *P2RY1* and *P2RY2* conferred better overall survival benefit to the patients as the cancers resembled more ‘normal tissue’ receptor expression. Greater activation of these receptors could presumably be detrimental to the cancer progression. Correspondingly, our qPCR findings showed that the earlier stage cancer cell lines, H103 and SAS, had high expression of genes encoding P2Y_1_ and P2Y_2_ receptors compared to the later stage OSCC cell line, H376. We previously demonstrated that the poorly differentiated, nonkeratinizing, and advanced stage OSCC biopsies had weak immunohistochemical staining for P2Y_2_ receptor expression compared to the well-keratinizing and earlier stage OSCC (the related findings are reproduced in Figure S4 for readers’ convenience) [43]. Comparing with other cancer types, glioblastoma multiforme and acute myeloid leukemia showed a significant upregulation of *P2RY1* and *P2RY2*, respectively, compared to their normal tissues, and conversely, the low expression of the genes provided more advantage in the overall patient survival [37].

P2Y_1_, P2Y_2_, and P2Y_11_ receptors are known G_q/11_-coupled receptors that activate the downstream intracellular Ca^2+^ and ERK signaling. We further demonstrated that the treatment with a high concentration of ATP was able to induce the activation of ERK signaling, leading to a significant S-phase arrest and increase in apoptotic cells in SAS. Although ATP activated intracellular Ca^2+^ release and ERK signaling in all three cell lines, no and minimal S-phase arrest were observed in H103 and H376, respectively. The contribution of the specific subtypes of P2Y receptors cannot be clearly elucidated, but we propose that the distinct P2Y receptor profiles collectively could mediate the varied effects of ATP observed between the cell lines.

We also found that ATPγS, the nondegradable ATP, and adenosine promoted a similar level of S-phase arrest in SAS compared to ATP itself. This suggests that both direct ATP-responsive receptors of P2Y_1_, P2Y_2_, and P2Y_11_ and adenosine-responsive receptors could independently mediate the cell cycle arrest in SAS. Although the P2Y_1_ receptor has a higher affinity towards ADP (one of the degradation products of ATP) compared to ATP, ATP has a high affinity towards P2Y_2_ and P2Y_11_ receptors [44]. Another class of ATP-responsive receptors includes the P2X ionotropic receptors. Amongst the P2X_1-7_ receptors, the P2X_7_ receptor was also shown to activate ERK signaling in ovarian cancer cells [45]. However, the P2X_7_ receptor in general requires a high ATP concentration of more than 100 mM to be activated, and it has been reported that the expression of the P2X_7_ receptor in head and neck cancers is limited [46]. Unfortunately, the use of suramin as a general P2Y receptor antagonist in our study did not work well. Suramin is also a well-known DNA topoisomerase I and II inhibitor that could alone induce S-phase arrest [47]. Overall, these data support our hypothesis that the ATP-mediated ERK signaling was potentially a P2Y receptor-mediated effect especially in SAS cells.

In esophageal, pancreatic, and colorectal adenocarcinoma cell lines, activation of ATP at the micromolar range or its direct receptor subtype agonist on P2Y_1_ and P2Y_2_ receptors promoted S-phase arrest of the cell cycle, accompanied by a variable increase in apoptotic cell number [48,49]. Another pancreatic cancer cell study reported a decrease in cell proliferation with S-phase cell cycle arrest [50]. The latter finding was, however, in contrast to our previous report on SAS that ATP incubation slightly increased cell proliferation using the same BrDU assay [43]. Therefore, our finding that showed the S-phase arrest in SAS could produce an increase in apoptotic cell number correlated well with other reported studies.

ERK signaling has been well documented as one of the downstream signalings of P2Y receptors and also to mediate cell death in a cancer-specific manner [51]. It has been reported that the transient ERK activation by ADP and ATP via the P2Y_1_ receptor caused apoptosis in astrocytoma cells [52]. A similar observation was made in prostate cancer cells whereby the treatment with an ADP analog induced P2Y_1_ receptor-mediated apoptosis effect via ERK activation [53]. We also propose that the S-phase cell cycle arrest in SAS could be due to an upregulation of downstream signaling molecules of ERK signaling, such as a cyclin-dependent kinase inhibitor, p21. ERK signaling activation has been well reported to be sufficient to promote the expression of p21 [54]. This could be studied further in the future.

Additionally, we also hypothesized that activation of P2Y receptors by the high ATP concentration in the tumor microenvironment may influence the efficacy of chemotherapeutic agents. Indeed, in breast cancer, ATP stimulation on the P2Y_2_ receptor could induce chemoresistance towards a variety of chemotherapeutic agents, including cisplatin, doxorubicin, paclitaxel, and gemcitabine, via indirect activation of IL-6 to trigger its downstream JAK-1-SOX9 signaling [55]. In a previous study, we reported that the efficacy of cisplatin was reduced selectively by ATP in H376, but not in H103 or SAS [43]. The mechanistic details of the differential effects between cells are yet to be understood fully, and it remains unclear whether these observations were due to an alteration in P2Y receptor signaling and/or altered expression of P2Y receptor subtypes in cancer. Further studies are required to prove the possible causal mechanisms. In this study, we attempted to elucidate if ATP could also influence the effect of docetaxel, a microtubule-stabilizing agent that causes a G2/M-phase arrest. For an enhanced treatment efficacy effect, it was expected that the combination treatment of ATP with docetaxel could increase the proportion of both S-phase and G2/M–phase cells when compared to docetaxel alone. However, preincubation with ATP did not shift the cell cycle distribution of the cells treated with docetaxel. This suggests that the effect of docetaxel alone was stronger and ATP did not influence the mitotic arrest effect of docetaxel.

## 5. Conclusions

In this study, we suggest that the stimulation with ATP may lead to the activation of P2Y receptors and further activate the ERK signaling for apoptosis in an OSCC cancer cell-specific manner. A possible explanation may lie in the distinct purinergic receptor profiles of the cells, which are yet to be confirmed. It is crucial to pinpoint that the direct effects of ATP in OSCC cell lines via P2Y receptors remain elusive and have not been further confirmed in this study by in-depth mechanistic studies. In our present work, the use of a general receptor antagonist was unable to demonstrate the direct activation of a specific P2Y receptor leading to S-phase arrest. In the future, the use of specific antagonist receptor subtypes or P2Y receptor subtypes in knockout and knockdown studies through gene editing and transfection, respectively, could validate the role of the receptors in OSCC. Further, the application of multiple study models, such as the three-dimensional in vitro tumor-tissue invasion model, animal models, and human explants, could be used to further strengthen our proposed hypothesis [56,57,58]. Overall, these preliminary findings highlight the potential role of the cancer-specific landscape of P2Y receptors in OSCC in influencing the antitumor effect induced by ATP. These findings may further help us to understand the role of ATP in OSCC cells with distinct profiles of purinergic receptors for the development of a tissue-specific therapy in OSCC.

## Data Availability

The data presented in this study are openly available in FigShare (doi:10.6084/m9.figshare.c.5614346).

## Supplementary Materials

The following are available online at https://www.mdpi.com/article/10.3390/life11111170/s1, Figure S1: Raw outputs of the expression of p-ERK and total ERK proteins from Western blotting of SAS cell lines, Figure S2: Raw outputs of the expression of p-ERK and total ERK proteins from Western blotting of H103 cell lines, Figure S3: Raw outputs of the expression of p-ERK and total ERK proteins from Western blotting of H376 cell lines, Figure S4: Immunohistochemistry staining of P2Y2 receptor in OSCC patient samples, Table S1: Clinicopathological characteristics of OSCC cell lines, Table S2: Primer sequences for qPCR analysis,: Table S3: Densitometry readings of p-ERK protein bands from Western blotting of SAS cell lines, Table S4: Densitometry readings of p-ERK protein bands from Western blotting of H103 cell lines, Table S5: Densitometry readings of p-ERK protein bands from Western blotting of H376 cell lines.

## References

[1] Luo Y., Liu F., Guo J., Gui R. (2020). Upregulation of circ_0000199 in circulating exosomes is associated with survival outcome in OSCC. Sci. Rep..

[2] Ishida K., Tomita H., Nakashima T., Hirata A., Tanaka T., Shibata T., Hara A. (2017). Current mouse models of oral squamous cell carcinoma: Genetic and chemically induced models. Oral Oncol..

[3] Economopoulou P., de Bree R., Kotsantis I., Psyrri A. (2019). Diagnostic tumor markers in head and neck squamous cell carcinoma (HNSCC) in the clinical setting. Front. Oncol..

[4] Pentangelo G., Nisticò S.P., Provenzano E., Cisale G.Y., Bennardo L. (2021). Topical 5% Imiquimod Sequential to Surgery for HPV-Related Squamous Cell Carcinoma of the Lip. Medicina.

[5] Bennardo L., Bennardo F., Giudice A., Passante M., Dastoli S., Morrone P., Provenzano E., Patruno C., Nisticò S.P. (2021). Local chemotherapy as an adjuvant treatment in unresectable squamous cell carcinoma: What do we know so far?. Curr. Oncol..

[6] Brands M.T., Smeekens E.A., Takes R.P., Kaanders J.H., Verbeek A.L., Merkx M.A., Geurts S.M. (2019). Time patterns of recurrence and second primary tumors in a large cohort of patients treated for oral cavity cancer. Cancer Med..

[7] Hakim S.G., Von Bialy R., Falougy M., Steller D., Tharun L., Rades D., Sieg P., Alsharif U. (2021). Impact of stratified resection margin classification on local tumor control and survival in patients with oral squamous cell carcinoma. J. Surg. Oncol..

[8] Wang W., Adeoye J., Thomson P., Choi S.W. (2021). Multiple tumour recurrence in oral, head and neck cancer: Characterising the patient journey. J. Oral Pathol. Med..

[9] Wu H.-T., Chen W.-T., Li G.-W., Shen J.-X., Ye Q.-Q., Zhang M.-L., Chen W.-J., Liu J. (2020). Analysis of the Differentially Expressed Genes Induced by Cisplatin Resistance in Oral Squamous Cell Carcinomas and Their Interaction. Front. Genet..

[10] Law Z.-J., Khoo X.-H., Lim P.-T., Ming L.C., Goh B.H., Lee W.-L. (2021). Extracellular Vesicle-Mediated Chemoresistance in Oral Squamous Cell Carcinoma. Front. Mol. Biosci..

[11] Huang Z., Xie N., Illes P., Di Virgilio F., Ulrich H., Semyanov A., Verkhratsky A., Sperlagh B., Yu S.-G., Huang C. (2021). From purines to purinergic signalling: Molecular functions and human diseases. Signal Transduct. Target. Ther..

[12] Burnstock G. (2006). Purinergic signalling. Br. J. Pharmacol..

[13] Burnstock G. (2020). Introduction to purinergic signaling. Purinergic Signaling.

[14] Campos-Contreras A.d.R., Díaz-Muñoz M., Vázquez-Cuevas F.G. (2020). Purinergic signaling in the hallmarks of cancer. Cells.

[15] Patergnani S., Danese A., Bouhamida E., Aguiari G., Previati M., Pinton P., Giorgi C. (2020). Various aspects of calcium signaling in the regulation of apoptosis, autophagy, cell proliferation, and cancer. Int. J. Mol. Sci..

[16] Pellegatti P., Raffaghello L., Bianchi G., Piccardi F., Pistoia V., Di Virgilio F. (2008). Increased level of extracellular ATP at tumor sites: In vivo imaging with plasma membrane luciferase. PLoS ONE.

[17] Di Virgilio F., Adinolfi E. (2017). Extracellular purines, purinergic receptors and tumor growth. Oncogene.

[18] Vultaggio-Poma V., Sarti A.C., Di Virgilio F. (2020). Extracellular ATP: A feasible target for cancer therapy. Cells.

[19] Feng L.-L., Cai Y.-Q., Zhu M.-C., Xing L.-J., Wang X. (2020). The yin and yang functions of extracellular ATP and adenosine in tumor immunity. Cancer Cell Int..

[20] Khalid M., Brisson L., Tariq M., Hao Y., Guibon R., Fromont G., Mortadza S.A.S., Mousawi F., Manzoor S., Roger S. (2017). Carcinoma-specific expression of P2Y11 receptor and its contribution in ATP-induced purinergic signalling and cell migration in human hepatocellular carcinoma cells. Oncotarget.

[21] Ding Y., Liu P., Zhang S., Tao L., Han J. (2018). Screening pathogenic genes in oral squamous cell carcinoma based on the mRNA expression microarray data. Int. J. Mol. Med..

[22] Suwanwela J., Osathanon T. (2017). Inflammation related genes are upregulated in surgical margins of advanced stage oral squamous cell carcinoma. J. Oral Biol. Craniofacial Res..

[23] Bellefeuille S.D., Molle C.M., Gendron F.-P. (2019). Reviewing the role of P2Y receptors in specific gastrointestinal cancers. Purinergic Signal..

[24] Le H.T.T., Murugesan A., Ramesh T., Yli-Harja O., Saravanan K.M., Kandhavelu M. (2021). Molecular interaction of HIC, an agonist of P2Y1 receptor, and its role in prostate cancer apoptosis. Int. J. Biol. Macromol..

[25] Avanzato D., Genova T., Pla A.F., Bernardini M., Bianco S., Bussolati B., Mancardi D., Giraudo E., Maione F., Cassoni P. (2016). Activation of P2X7 and P2Y11 purinergic receptors inhibits migration and normalizes tumor-derived endothelial cells via cAMP signaling. Sci. Rep..

[26] Vinette V., Placet M., Arguin G., Gendron F.-P. (2015). Multidrug resistance-associated protein 2 expression is upregulated by adenosine 5’-triphosphate in colorectal cancer cells and enhances their survival to chemotherapeutic drugs. PLoS ONE.

[27] Eun S.Y., Ko Y.S., Park S.W., Chang K.C., Kim H.J. (2015). P2Y2 nucleotide receptor-mediated extracellular signal-regulated kinases and protein kinase C activation induces the invasion of highly metastatic breast cancer cells. Oncol. Rep..

[28] Li W., Qiu Y., Zhang H., Liu Y., You J., Tian X., Fang W. (2013). P2Y2 receptor promotes cell invasion and metastasis in prostate cancer cells. Br. J. Cancer.

[29] Chadet S., Jelassi B., Wannous R., Angoulvant D., Chevalier S., Besson P., Roger S. (2014). The activation of P2Y2 receptors increases MCF-7 breast cancer cells migration through the MEK-ERK1/2 signalling pathway. Carcinogenesis.

[30] Zaparte A., Cappellari A.R., Brandao C.A., de Souza J.B., Borges T.J., Kist L.W., Bogo M.R., Zerbini L.F., Pinto L.F.R., Glaser T. (2021). P2Y2 receptor activation promotes esophageal cancer cells proliferation via ERK1/2 pathway. Eur. J. Pharmacol..

[31] Limami Y., Pinon A., Leger D.Y., Mousseau Y., Cook-Moreau J., Beneytout J.-L., Delage C., Liagre B., Simon A. (2011). HT-29 colorectal cancer cells undergoing apoptosis overexpress COX-2 to delay ursolic acid-induced cell death. Biochimie.

[32] Limami Y., Pinon A., Leger D.Y., Pinault E., Delage C., Beneytout J.-L., Simon A., Liagre B. (2012). The P2Y2/Src/p38/COX-2 pathway is involved in the resistance to ursolic acid-induced apoptosis in colorectal and prostate cancer cells. Biochimie.

[33] Woods L.T., Jasmer K.J., Forti K.M., Shanbhag V.C., Camden J.M., Erb L., Petris M.J., Weisman G.A. (2020). P2Y2 receptors mediate nucleotide-induced EGFR phosphorylation and stimulate proliferation and tumorigenesis of head and neck squamous cell carcinoma cell lines. Oral Oncol..

[34] Mishima K., Inoue K., Hayashi Y. (2002). Overexpression of extracellular-signal regulated kinases on oral squamous cell carcinoma. Oral Oncol..

[35] Kordi-Tamandani D., Sabers E., Jamali S., Rigi Ladiz M. (2014). ERK and RAF1 genes: Analysis of methylation and expression profiles in patients with oral squamous cell carcinoma. Br. J. Biomed. Sci..

[36] Prime S.S., Nixon S.V., Crane I.J., Stone A., Matthews J.B., Maitland N.J., Remnant L., Powell S.K., Game S.M., Scully C. (1990). The behaviour of human oral squamous cell carcinoma in cell culture. J. Pathol..

[37] Tang Z., Li C., Kang B., Gao G., Li C., Zhang Z. (2017). GEPIA: A web server for cancer and normal gene expression profiling and interactive analyses. Nucleic Acids Res..

[38] Jin H., Eun S.Y., Lee J.S., Park S.W., Lee J.H., Chang K.C., Kim H.J. (2014). P2Y 2 receptor activation by nucleotides released from highly metastatic breast cancer cells increases tumor growth and invasion via crosstalk with endothelial cells. Breast Cancer Res..

[39] Abbracchio M.P., Burnstock G., Boeynaems J.-M., Barnard E.A., Boyer J.L., Kennedy C., Knight G.E., Fumagalli M., Gachet C., Jacobson K.A. (2006). International Union of Pharmacology LVIII: Update on the P2Y G protein-coupled nucleotide receptors: From molecular mechanisms and pathophysiology to therapy. Pharmacol. Rev..

[40] Mansfield K.J., Hughes J.R. (2014). P2Y receptor modulation of ATP release in the urothelium. BioMed Res. Int..

[41] Kita M., Ano Y., Inoue A., Aoki J. (2019). Identification of P2Y receptors involved in oleamide-suppressing inflammatory responses in murine microglia and human dendritic cells. Sci. Rep..

[42] Ostruszka L.J., Leu K.M., Baker L.H., Shewach D.S. (2004). Docetaxel Enhances S-Phase Cytotoxicity with dFdCyd Resulting in Synergy.

[43] Law L.M., Azmi N., Paterson I.C., Ng P.Y. P2Y Purinergic Receptor Signaling in Oral Squamous Cell Carcinoma Cell Line Sand Its Role in Proliferation and Cisplatin-Mediated Apoptosis. *Sains Malaysiana*
**2022**, *51*. https://www.ukm.my/jsm/english_journals/vol51num1_2022/contentsVol51num1_2022.html.

[44] Abbracchio M.P., Boeynaems J.M., Boyer J.L., Burnstock G., Ceruti S., Fumagalli M., Gachet C., Hills R., Humphries R.G., Inoue K. (2019). P2Y receptors (version 2019.4) in the IUPHAR/BPS Guide to Pharmacology Database. IUPHAR/BPS Guide Pharmacol. CITE.

[45] Vázquez-Cuevas F.G., Martínez-Ramírez A.S., Robles-Martínez L., Garay E., García-Carrancá A., Montiel M.D.P., Castañeda-García C., Arellano R.O. (2014). Paracrine stimulation of P2X7 receptor by ATP activates a proliferative pathway in ovarian carcinoma cells. J. Cell. Biochem..

[46] Alves L.A., de Melo Reis R.A., de Souza C.A.M., de Freitas M.S., Teixeira P.C.N., Ferreira D.N.M., Xavier R.F. (2014). The P2X7 receptor: Shifting from a low-to a high-conductance channel—An enigmatic phenomenon?. Biochim. Biophy. Acta (BBA) Biomembr..

[47] Bojanowski K., Lelievre S., Markovits J., Couprie J., Jacquemin-Sablon A., Larsen A.K. (1992). Suramin is an inhibitor of DNA topoisomerase II in vitro and in Chinese hamster fibrosarcoma cells. Proc. Natl. Acad. Sci. USA.

[48] Rapaport E. (1983). Treatment of human tumor cells with ADP or ATP yields arrest of growth in the S phase of the cell cycle. J. Cell. Physiol..

[49] Maaser K., Hopfner M., Kap H., Sutter A.P., Barthel B., Von Lampe B., Zeitz M., Scherubl H. (2002). Extracellular nucleotides inhibit growth of human oesophageal cancer cells via P2Y2-receptors. Br. J. Cancer.

[50] Yamada T., Okajima F., Akbar M., Tomura H., Narita T., Yamada T., Ohwada S., Morishita Y., Kondo Y. (2002). Cell cycle arrest and the induction of apoptosis in pancreatic cancer cells exposed to adenosine triphosphate in vitro. Oncol. Rep..

[51] Pathak R., Bhatnagar S., Dubey A.K. (2008). Mechanisms underlying the opposing effects of P2Y receptors on the cell cycle. J. Recept. Signal Transduct..

[52] Sellers L.A., Simon J., Lundahl T.S., Cousens D.J., Humphrey P.P.A., Barnard E.A. (2001). Adenosine Nucleotides Acting at the Human P2Y1Receptor Stimulate Mitogen-activated Protein Kinases and Induce Apoptosis. J. Biol. Chem..

[53] Wei Q., Costanzi S., Liu Q.-Z., Gao Z.-G., Jacobson K. (2011). Activation of the P2Y1 receptor induces apoptosis and inhibits proliferation of prostate cancer cells. Biochem. Pharmacol..

[54] Bottazzi M.E., Zhu X., Böhmer R.M., Assoian R.K. (1999). Regulation of P21cip1 Expression by Growth Factors and the Extracellular Matrix Reveals a Role for Transient ERK Activity in G1 Phase. J. Cell Biol..

[55] Yang H., Geng Y.H., Wang P., Yang H., Zhou Y.T., Zhang H.Q., He H.Y., Fang W.G., Tian X.X. (2020). Extracellular ATP promotes breast cancer invasion and chemoresistance via SOX9 signaling. Oncogene.

[56] Salo T., Sutinen M., Apu E.H., Sundquist E., Cervigne N.K., De Oliveira C.E., Akram S.U., Ohlmeier S., Suomi F., Eklund L. (2015). A novel human leiomyoma tissue derived matrix for cell culture studies. BMC Cancer.

[57] Apu E.H., Akram S.U., Rissanen J., Wan H., Salo T. (2018). Desmoglein 3—Influence on oral carcinoma cell migration and invasion. Exp. Cell Res..

[58] Demers I., Donkers J., Kremer B., Speel E.J. (2020). Ex Vivo Culture Models to Indicate Therapy Response in Head and Neck Squamous Cell Carcinoma. Cells.

