# Supplementary material for: Extracellular ATP Induced S-Phase Cell Cycle Arrest via P2Y Receptor-Activated ERK Signaling in Poorly Differentiated Oral Squamous Cell Carcinoma SAS Cells
Source: Life (Basel). 2021 Nov 2;11(11):1170. doi: 10.3390/life11111170 (PMC8624497; doi:10.3390/life11111170)
Supplement: Supplementary file 1 [file life-11-01170-s001.zip › life-1400831-supplementary.pdf]

life

MDPI

Supplementary Materials

Table S1. Clinicopathological characteristics of OSCC cell lines.

Cell lines	Nationality	Age	Sex	Site[a]	Nodal metastasis (N)	Distant metastasis (M)	Pathology[b] (P)	STNMP Grade[c]
SAS	Japanese	69	Female	T	+	−	P	II
H103	British	32	Male	T	−	−	W	I
H376	British	40	Female	FOM	+	−	W	III

[a] Site: T, tongue; FOM, floor of mouth.
[b] Pathology: W, well-differentiated; P, poorly differentiated.
[c] STNMP Grade: Prognostic indicator of OSCC with 51.5 %, 40.7 % and 21.6 % of 5-year survival for patients with stage I, II, and III respectively.

Table S2. Primers sequence for qPCR analysis.

Gene		Sequence (5'-3')
P2YR1	Forward	CGGTCCGGGTTCGTCCT
	Reverse	TAGTAAAACTGGAAGCCCGTCTTG
P2YR2	Forward	CTCTACTTTGTCACCACCAGCGC
	Reverse	CCACGAAGCGGCTGAAGA
P2YR11	Forward	CAGCGTCATCTTCATCACCTGC
	Reverse	AGGTGGCTTCGGGCGA
TUBA6	Forward	CCGGGCAGTGTTTGTAGACT
	Reverse	TTGCCTGTGATGAGTTGCTC
RPS13	Forward	CAGTCGGCTTTACCCTATCG
	Reverse	CCCTTCTTGGCCAGTTTGTA

Table S3. Densitometry analysis of p-ERK in ATP-treated SAS cell line, $n = 3$.

Experiment 1, n1					
Time	Vol. %	Volume	Height	Area	Vol/Vol
Untreated	9.381	4540511	11348	1360	1
5 mins	11.668	5647779	14404	1394	1.243864
10 mins	15.794	7644492	17291	1496	1.683619
15 mins	14.341	6941499	16080	1462	1.528792
30 mins	15.97	7729771	18119	1462	1.702401
45 mins	14.14	6843979	17163	1462	1.507315
60 mins	10.47	5067685	14250	1428	1.116105
75 mins	8.237	3987008	11575	1428	0.878097
90 mins	6.468	3130558	9497	1394	0.689473
120 mins	4.43	2144351	7742	1394	0.472271
Experiment 2, n2					
Time	Vol. %	Volume	Height	Area	Vol/Vol
Untreated	5.828	1846348	4458	1440	1
5 mins	9.113	2887278	7247	1440	1.563778
10 mins	15.33	4856682	10418	1620	2.630426
15 mins	16.536	5238948	10461	1620	2.837465
30 mins	18.603	5893780	11654	1620	3.192128
45 mins	15.016	4757280	10137	1548	2.576589
60 mins	9.621	3047988	6688	1620	1.65082
75 mins	9.954	3153526	8535	1440	1.70798

Time	Vol. %	Volume	Height	Area	Vol/Vol
90 mins	11.865	3759038	9657	1404	2.035931
120 mins	6.442	2040935	5517	1368	1.10539
**Experiment 3, n3**					
**Time**	**Vol. %**	**Volume**	**Height**	**Area**	**Vol/Vol**
Untreated	9.529	3221704	8288	2262	1
5 mins	18.808	6359015	14427	2320	1.973805
10 mins	18.413	6225569	12756	2842	1.932384
15 mins	15.45	5223609	12180	2378	1.621381
30 mins	13.602	4598722	10948	2378	1.427419
45 mins	12.967	4384008	10043	2552	1.360773
60 mins	6.746	2280877	5530	2494	0.707972
75 mins	4.485	1516444	4019	2378	0.470696
90 mins	5.867	1983564	5366	2146	0.615688
120 mins	4.321	1460808	4566	2204	0.453427

Table S4. Densitometry analysis of p-ERK in ATP-treated H103 cell line, $n = 3$.

**Experiment 1, n1**					
**Time**	**Vol. %**	**Volume**	**Height**	**Area**	**Vol/Vol**
Untreated	1.95	852070	1448	3060	1
5 mins	20.245	8847329	10259	2992	10.38334
10 mins	11.019	4815358	7139	2992	5.651364
15 mins	12.231	5344982	6543	2992	6.272938
30 mins	15.085	6592279	7672	2992	7.736781
45 mins	9.187	4015041	4964	2924	4.712102
60 mins	8.621	3767402	4373	2924	4.42147
75 mins	6.973	3047145	3820	2856	3.576167
90 mins	7.332	3204022	3898	2924	3.76028
120 mins	0.804	351176	1421	2924	0.412145
**Experiment 2, n2**					
**Time**	**Vol. %**	**Volume**	**Height**	**Area**	**Vol/Vol**
Untreated	5.645	761129	1068	2860	1
5 mins	21.051	2838164	4429	2860	3.728887
10 mins	19.824	2672776	3315	2860	3.511594
15 mins	10.944	1475461	2027	2860	1.938516
30 mins	9.505	1281474	1771	2860	1.683649
45 mins	8.227	1109133	1104	2860	1.457221
60 mins	5.945	801497	1047	2925	1.053037
75 mins	9.506	1281608	1290	2990	1.683825
90 mins	6.05	815626	1295	2600	1.0716
120 mins	1.979	266793	633	2795	0.350523
**Experiment 3, n3**					
**Time**	**Vol. %**	**Volume**	**Height**	**Area**	**Vol/Vol**
Untreated	8.007	514022	658	2924	1
5 mins	22.812	1464427	1858	2788	2.848958
10 mins	10.464	671740	696	2788	1.306831
15 mins	11.184	717977	899	2652	1.396783
30 mins	9.349	600176	764	2924	1.167608
45 mins	9.309	597555	534	2924	1.162509
60 mins	5.994	384775	497	2788	0.748557
75 mins	7.206	462589	480	2788	0.89994

90 mins	7.241	464818	522	2448	0.904276
120 mins	3.53	226628	381	2720	0.440892

Table S5. Densitometry analysis of p-ERK in ATP-treated H376 cell line, *n* = 3.

**Experiment 1, n1**					
**Time**	**Vol. %**	**Volume**	**Height**	**Area**	**Vol/Vol**
Untreated	100	1411494	4142	2205	1
5 mins	393.576	5555299	11926	2961	3.935758
10 mins	291.708	4117439	10325	2520	2.917079
15 mins	220.342	3110119	7735	2646	2.203423
30 mins	201.231	2840361	6754	2583	2.012308
45 mins	147.575	2083009	5037	2331	1.475748
60 mins	126.107	1779992	4165	2394	1.261069
75 mins	212.977	3006162	5953	2709	2.129773
90 mins	196.409	2772308	5400	2772	1.964095
120 mins	177.194	1693711	3507	2880	1.199942
**Experiment 2, n2**					
**Time**	**Vol. %**	**Volume**	**Height**	**Area**	**Vol/Vol**
Untreated	100	955850	2260	2432	1
5 mins	368.585	3523119	11220	2432	3.685849
10 mins	436.385	4171188	10020	3008	4.363852
15 mins	132.404	1265581	2626	2944	1.324037
30 mins	508.098	4856650	7777	2944	5.080975
45 mins	240.55	2299296	6912	2496	2.405499
60 mins	203.998	1949918	3860	2432	2.039983
75 mins	176.91	1690990	3518	2304	1.769096
90 mins	137.834	1317482	2346	2624	1.378336
120 mins	177.194	1693711	3507	2880	1.771942
**Experiment 3, n3**					
**Time**	**Vol. %**	**Volume**	**Height**	**Area**	**Vol/Vol**
Untreated	100	1047049	2719	1677	1
5 mins	303.621	3179060	7858	1935	3.036209
10 mins	236.068	2471748	6176	1849	2.36068
15 mins	117.672	1232080	3545	1806	1.176717
30 mins	197.01	2062792	5370	1892	1.970101
45 mins	138.202	1447040	4415	1892	1.382017
60 mins	92.085	964179	2835	1634	0.920854
75 mins	90.343	945936	2875	1892	0.90343
90 mins	99.969	1046724	2852	1591	0.99969
120 mins	56.374	590260	1494	1849	0.563737

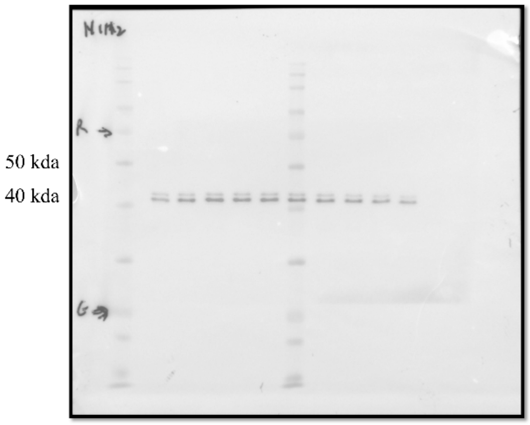

Representative of overlay blot of p-ERK with protein ladder

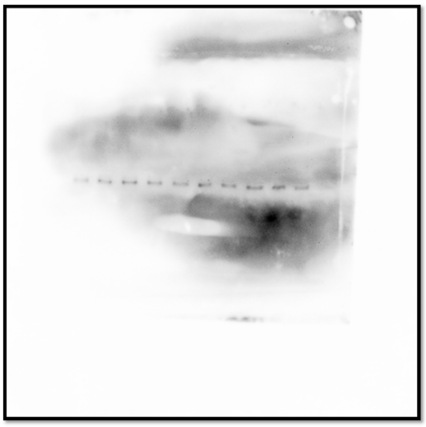

Original blot of total ERK

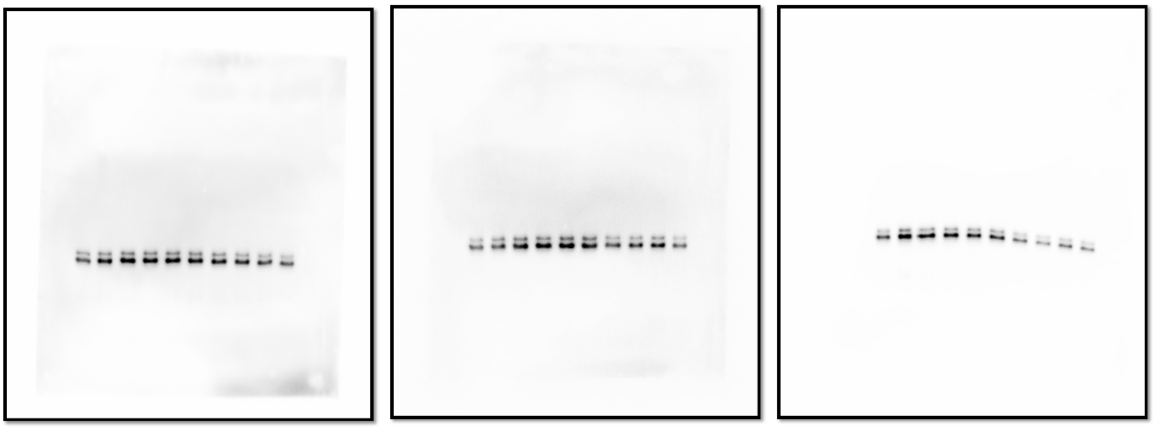

Original blot of p-ERK (n = 3)

Figure S1. Raw outputs of the expression of p-ERK and total ERK proteins from Western blotting**.** The original blot images of p-ERK in ATP-treated SAS cell line, $n = 3$.

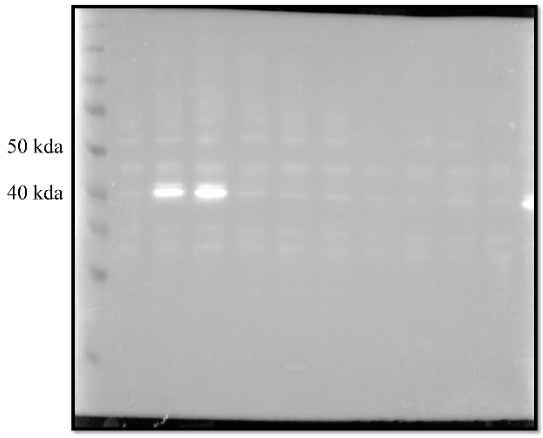

Representative of overlay blot of p-ERK with protein ladder

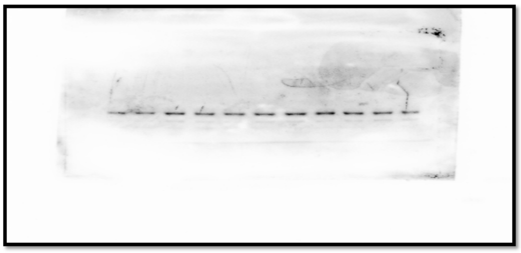

Original blot of total ERK

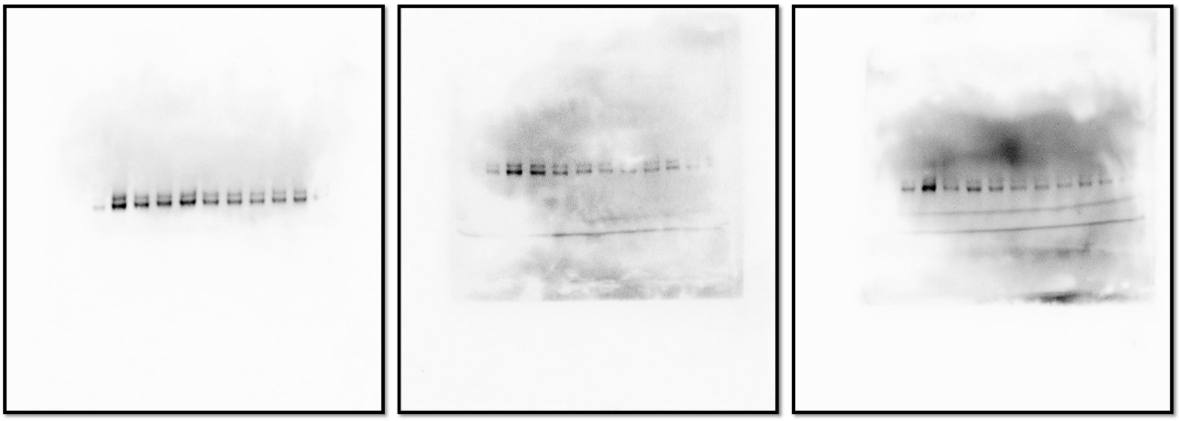

Original blot of p-ERK (n = 3)

Figure S2. Raw outputs of the expression of p-ERK and total ERK proteins from Western blotting**.** The original blot images of p-ERK in ATP-treated H103 cell line, $n = 3$.

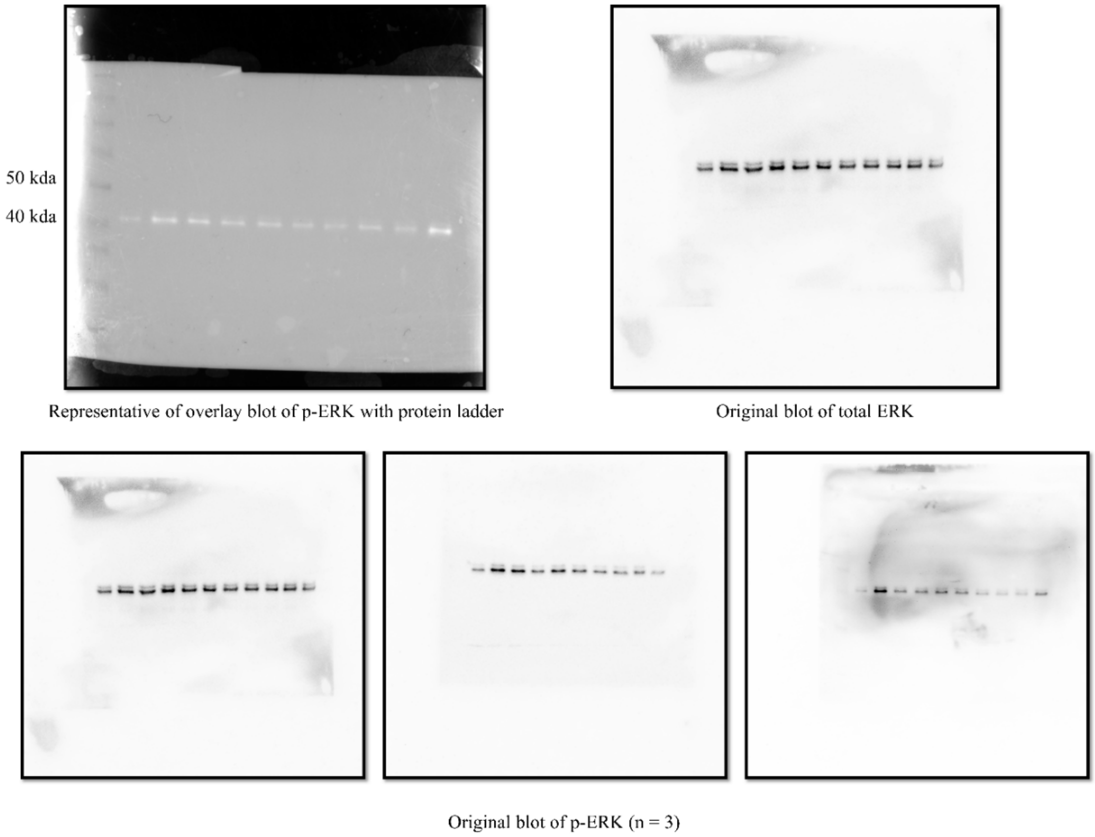

Figure S3. Raw outputs of the expression of p-ERK and total ERK proteins from Western blotting. The original blot images of p-ERK in ATP-treated H376 cell line, $n = 3$.

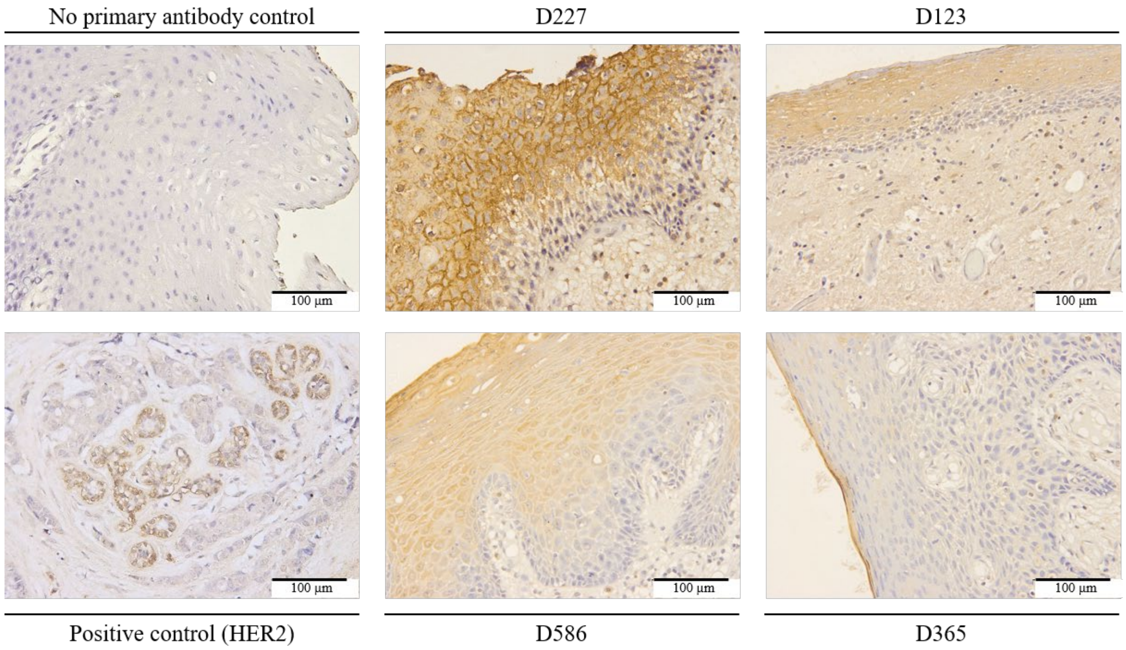

Figure S4. Immunohistochemistry staining of $P2Y_2$ receptor in OSCC patient samples. The keratinizing early stage OSCC (D227 and D586 samples) demonstrated strong cytoplasmic staining. Meanwhile, the poorly differentiated and non-keratinizing advanced stage OSCC (D123 sample) showed weak $P2Y_2$ staining. In addition, a loss of $P2Y_2$ staining was observed during the transition from normal epithelium to epithelial dysplasia (D365 sample). The representative negative control was the OSCC sample without antibody incubation. HER2 positive breast cancer tissue was used as the positive control. The figure is reproduced from a reported article for readers' convenience [43].
